# Clopidogrel Resistance in Patients With Stroke Recurrence Under Single or Dual Antiplatelet Treatment

**DOI:** 10.3389/fneur.2021.652416

**Published:** 2021-08-10

**Authors:** Hyun Goo Kang, Seung Jae Lee, Sung Hyuk Heo, Dae-il Chang, Bum Joon Kim

**Affiliations:** ^1^Department of Neurology, Research Institute of Clinical Medicine of Jeonbuk National University - Biomedical Research Institute of Jeonbuk National University Hospital, Jeonju, South Korea; ^2^Institute for Molecular Biology and Genetics and Department of Chemistry, Jeonbuk National University, Jeonju, South Korea; ^3^Department of Neurology, Kyung Hee University School of Medicine, Seoul, South Korea; ^4^Department of Neurology, Asan Medical Center, University of Ulsan, School of Medicine, Seoul, South Korea

**Keywords:** antiplatelet resistance, aspirin, clopidogrel, smoking, VerifyNow, prevention

## Abstract

**Background:** The factors associated with clopidogrel resistance in patients with stroke recurrence receiving single or dual antiplatelet treatment (SAPT or DAPT) may differ. This study compared the high on-treatment platelet reactivities (HPRs) and the factors associated with clopidogrel resistance in recurrent ischemic stroke patients receiving clopidogrel or aspirin and clopidogrel.

**Methods:** We enrolled and allocated 275 recurrent ischemic stroke patients to the clopidogrel and DAPT groups and compared their demographics, conventional risk factors, and P2Y12 reaction units (PRUs). Clopidogrel resistance was categorized as PRU higher than 275. We performed a multivariate logistic regression analysis to determine the factors underlying clopidogrel resistance during SAPT and DAPT.

**Results:** In total, 145 (52.7%) and 130 (47.3%) patients received clopidogrel and DAPT, respectively at recurrence. The risk factors of the two groups were not significantly different, except that coronary artery disease was more frequent in the DAPT group. The PRU was higher (255 ± 91 vs. 221 ± 84; *p* = 0.002) and clopidogrel resistance was more frequent (45.5 vs. 31.5%; *p* = 0.018) in the SAPT than in the DAPT group. Hyperlipidemia was associated with clopidogrel resistance during SAPT, and smoking (Odds ratio = 0.426, 95% confidence interval 0.210–0.861; *p* = 0.018) had a protective effect against clopidogrel resistance. For those receiving DAPT, old age, female, low hemoglobin A1c level, and high ARU were associated with clopidogrel resistance.

**Conclusions:** HPR and clopidogrel resistance were more frequent in recurrent ischemic stroke patients receiving clopidogrel than in those receiving DAPT. Smoking was independently associated with less clopidogrel resistance among those receiving clopidogrel SAPT but not in those receiving DAPT.

## Introduction

Antiplatelet treatment is one of the most important treatments for reducing non-cardioembolic ischemic stroke. However, a considerable proportion of patients still experience ischemic stroke recurrence during appropriate antiplatelet treatment. Various factors are involved in antiplatelet treatment failure. Approximately 20–30% of patients receiving antiplatelet treatment show high platelet reactivity (high on-treatment platelet reactivity; HPR) (1). Several factors affect HPR during clopidogrel treatment, including genetic variations and drug-drug interactions involving hepatic cytochrome P450.

As clopidogrel is a prodrug activated by the hepatic cytochrome P450, factors influencing the hepatic cytochrome P450 system may affect its response. Smoking, one of the major risk factors for ischemic stroke (2), also enhances the activity of the P450 system (3), which increases the efficacy of clopidogrel (smoker's paradox) (4, 5). Recently, a *post-hoc* analysis of the CHANCE (Clopidogrel in High-Risk Patients with Acute Non-Disabling Cerebrovascular Events) trial revealed the interaction between smoking status and the contribution of clopidogrel to the early recurrence of ischemic stroke (6). The incidence of stroke was lower in currently smoking than in non-smoking patients receiving treatment with aspirin and clopidogrel (dual antiplatelet treatment; DAPT).

However, the exact mechanism underlying the smoker's paradox observed in the previous study and the effect of smoking on the long-term use of clopidogrel in ischemic stroke patients was not verified. Furthermore, it is still unclear whether HPR is equally important in patients receiving long-term clopidogrel single antiplatelet treatment (SAPT) and DAPT (aspirin and clopidogrel). Here, we compared the HPR among patients who received clopidogrel SAPT and DAPT. The factors associated with clopidogrel resistance in recurrent ischemic stroke patients receiving clopidogrel SAPT and DAPT were investigated.

## Materials and Methods

### Patients

This was a retrospective study involving consecutively registered patients with acute ischemic stroke (within 7 days from stroke onset) confirmed by magnetic resonance imaging (MRI). All the patients were admitted to Kyung Hee University Hospital and Jeonbuk National University Hospital between January 2010 and December 2017. Patients who were receiving clopidogrel or aspirin and clopidogrel at the onset of the stroke due to a prior ischemic stroke were enrolled. The use of DAPT was based on the protocols of each center, and finally by the physicians' decision based on the risk of bleeding and recurrence of ischemia. Usually, DAPT is used for a short period after ischemic stroke, whereas for (1) those with concomitant coronary artery disease (CAD), (2) severe intra or extracranial cerebral artery stenosis, or (3) recurrent cardiovascular event under SAPT, DAPT was considered for a longer-duration. The duration of DAPT was also determined based on the physician's decision. Patients without clinical (i.e., history of prior use of antithrombotics unobtainable) or imaging (inappropriate to receive MRI) data and those without the results of VerifyNow tests were excluded.

All those who were admitted to the two centers and were receiving clopidogrel or aspirin and clopidogrel at the onset of stroke underwent routine VerifyNow P2Y12 tests or VerifyNow Aspirin and VerifyNow P2Y12 tests, respectively, on the day of admission to investigate the biochemical antiplatelet resistance. The use and adherence of any antithrombotics were investigated from the patient, caregiver or physicians prescribing any medication prior to stroke.

### Data Collection and Definition

We obtained the clinical and imaging data from a registry database and medical records, and we divided the enrolled patients into clopidogrel and DAPT (aspirin and clopidogrel) groups according to the antiplatelet treatment they received at the onset of the ischemic stroke recurrence. We investigated the factors associated with clopidogrel resistance in those receiving clopidogrel alone or in combination with aspirin. The patients who were smoking at the time of the study were categorized as smokers, whereas those who were not smoking or had stopped smoking for more than 1 year were categorized as non-smokers. We also reviewed the results of the laboratory tests and physical examination. These were results for hypertension, diabetes mellitus (DM), hyperlipidemia, and CAD, among others, which are the putative risk factors of cerebrovascular disease. Hypertension was defined as a case where 140/90 mmHg or more was found if it was checked while resting during admission. Hypertension was diagnosed by a previous history or measuring blood pressure after taking a break for 5 min or more when checking it at the hospital before discharge after the patients was stabilized. In case of suspicious white coat hypertension, the patient was recommended to write a blood pressure diary at home, and was considered at the first visit. DM was defined as a blood glucose level of >200 mg/dL for at least 2 h after an oral glucose challenge, fasting blood glucose level of >126 mg/dL, hemoglobin A1c (HbA1c) > 6.5%, or DM medication use (7). Hyperlipidemia was defined as venous low-density lipoprotein (LDL) cholesterol concentration of >160 mg/dL, total cholesterol (TC) of >240 mg/dL, and triglyceride (TG) >200 mg/dL (8). All three definitions were based on the levels after more than 12 h of fasting. CAD was established by CAD diagnosis by a cardiologist and CAD medication use or history of percutaneous coronary intervention or bypass surgery. The institutional review board of Jeonbuk National University Hospital approved this study (approval number: CUH 2020-01-008). We carried out all the procedures following the ethical standards of the institutional and national research committees and the Helsinki Declaration. Informed consent was waived due to the retrospective nature of the study.

### VerifyNow Aspirin and P2Y12 Assays

We used the VerifyNow P2Y12 assays to measure the aspirin reaction unit (ARU), the P2Y12 reaction unit (PRU) and the percentage inhibition of the platelet P2Y12 receptors. This method is based on the ability of activated platelets to bind to fibrinogen. It measures the changes in light transmittance to assess fibrinogen-mediated platelet aggregation in blood containing clopidogrel (9). The degree of aggregation is expressed as ARU for aspirin and PRU and the inhibition percentage for clopidogrel. A higher ARU value reflects greater arachidonic acid-induced platelet reactivity, and a higher PRU value reflects greater ADP-induced platelet activity. An ARU equal to or higher than 550 is defined as aspirin resistance. Because of the high prevalence of the CYP 2C19 variant in Korea, a PRU higher than 275 was predictive of clinical events. Therefore, in this study, a PRU higher than 275 was considered indicative of clopidogrel resistance (10–13).

### Statistical Analysis

First, we compared the demographics, clinical data, and HPR of the patients receiving clopidogrel and DAPT. We used Pearson's chi-squared or Fisher's exact test for categorical variables and student's *t*-test for continuous variables. The normality of distribution was tested and variables not showing normal distribution were test with Mann-Whitney *U* test and was presented as mean and interquartile ranges. Second, we performed a multivariate analysis to determine the independent factors associated with clopidogrel resistance in patients who received clopidogrel alone or in combination with aspirin. To avoid variable selection caused by spurious correlations, we included only the variables that were potentially associated with clopidogrel resistance (*p* <0.1) on univariate analysis as potential factors associated with clopidogrel resistance for the multivariate logistic regression model. Factors associated with PRU were also investigated using a multivariable analysis with linear regression model. The correlations between age and the ARU and PRU levels were investigated using the Pearson correlation coefficient. We set statistical significance at *p* < 0.05 (two-tailed). We used SPSS 21.0 (IBM Corporation, Armonk, NY) to perform all the statistical analyses.

## Results

**Figure 1:**
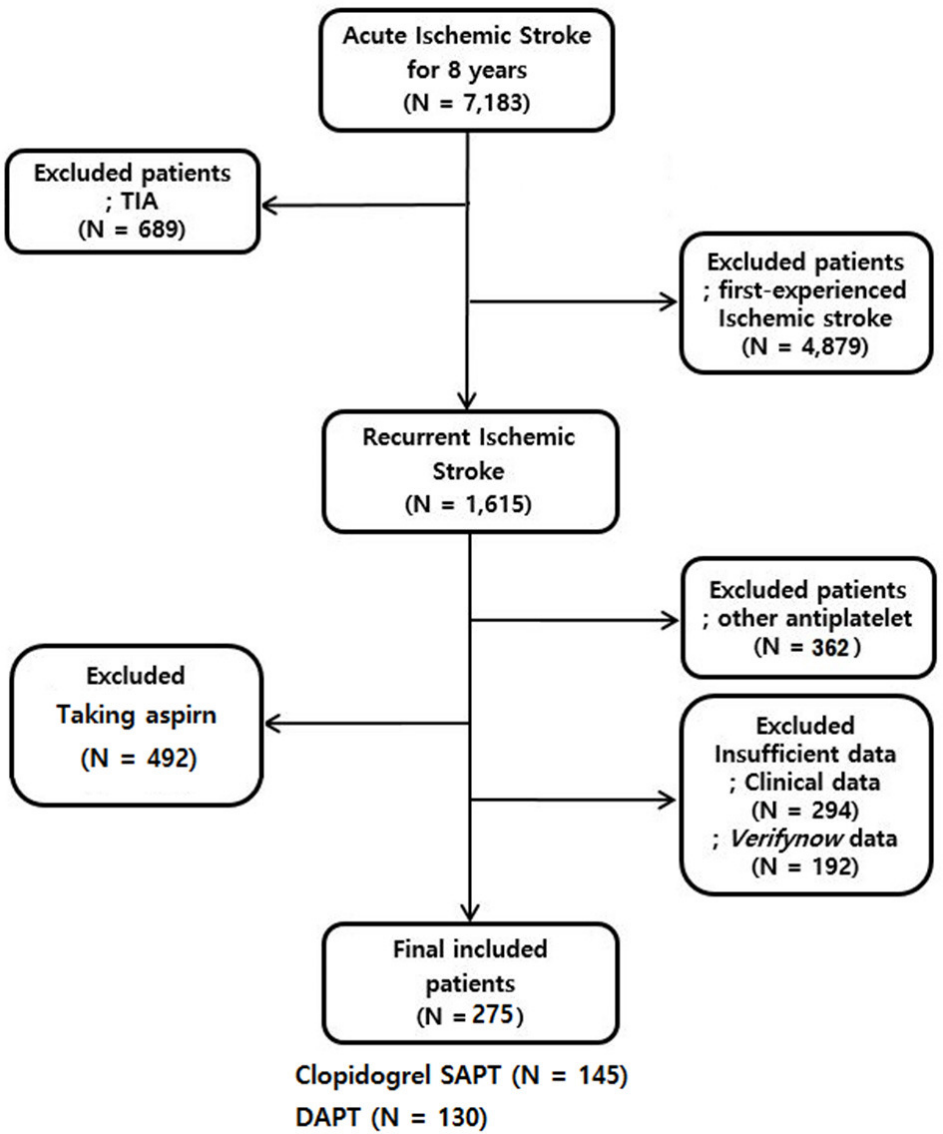
Selection of recurrent ischemic stroke patients who received clopidogrel or DAPT. TIA indicates a transient ischemic attack.

In total, 7,183 patients with onsets of acute ischemic strokes and transient ischemic attacks (TIA) within the previous 7 days were hospitalized and registered in the database. After excluding those with TIA (689 patients) and first stroke experience (4,879 patients), we identified 1,615 participants as recurrent ischemic stroke patients. Of them, 492 patients were taking aspirin and 362 were taking other antiplatelet agents or not taking an antiplatelet agent. Additionally, we excluded patients who did not have MR image or with poor image quality (*n* = 198), had limited clinical data (*n* = 96) and patients without VerifyNow data (*n* = 192). Consequently, the study evaluated the data of 275 recurrent ischemic stroke patients (Figure 1). The mean age of the enrolled patients was 70.2 ± 10.2 years-old, and 166 (60.4%) of them were males.

### HPR Among the Clopidogrel and DAPT Groups

The subjects were treated with clopidogrel (*n* = 145; 52.7%) or DAPT (*n* = 130; 47.3%) at the time of stroke recurrence. There were no significant differences between the demographics or risk factors of the two groups, except that the prevalence of previous CAD was higher in those receiving DAPT (50.6%) than in those receiving clopidogrel (23.9%). Patients taking clopidogrel showed significantly higher PRU values than those in the DAPT group (255 ± 91 vs. 221 ± 84; *p* = 0.002). The proportion of patients with clopidogrel resistance was also higher in those with recurrent stroke using clopidogrel than those using DAPT (45.5 vs. 31.5%; *p* = 0.018, Table 1).

**Table 1:** Clinical characteristics and HPR stratified by antiplatelet treatment for stroke recurrence.

	**Clopidogrel (*n* = 145)**	**DAPT (*n* = 130)**	***P-value***
Age, years	69.7 (10.9)	70.7 (9.4)	0.410
Male	87 (60.0)	79 (60.8)	0.896
Hypertension	131 (90.3)	108 (83.1)	0.074
Diabetes mellitus	68 (46.9)	63 (48.5)	0.795
Hyperlipidemia	102 (71.3)	103 (79.2)	0.132
Smoking	61 (42.1)	56 (43.1)	0.866
History of CAD^*^	16 (23.9)	40 (50.6)	0.001
Body-mass index (Kg/m^2^)	25 (3.8)	24 (3.5)	0.335
Laboratory results
C-reactive protein (mg/L)	0.14 [0.04–0.40]	0.23 [0.05–0.73]	0.153
Hemoglobin A1c (%)	6.6 (1.4)	6.9 (1.6)	0.082
Antiplatelet resistance
Aspirin reaction unit	NA	444 (75)	NA
P2Y12 reaction unit (base)	293 (64)	291 (61)	0.762
P2Y12 reaction unit	255 (91)	221 (84)	0.002
Percent inhibition	13 (26)	22 (29)	0.003
Clopidogrel resistance (%)	66 (45.5)	41 (31.5)	0.018
NIHSS score (initial)	4 (3–5)	4 (2-5)	0.155
NIHSS score (discharge)	3 (2-5)	3 (1-4)	0.003
mRS (discharge)	2 (1-3)	2 (1-3)	0.289

*Results are expressed as number (% column), mean (SD), or median (25–75 percentile range).*

*Non-parametric test was performed for continuous variables not showing normal distribution and presented as median (25–75 percentile range).*

*HPR, high on-treatment platelet reactivity; DAPT, dual antiplatelet treatment; CAD, coronary artery disease; NIHSS, National Institute of Stroke Scale; mRS, modified Rankin Scale.*

* *History of CAD: Clopidogrel (n = 67), DAPT (n = 79)*.

### Factors Associated With Clopidogrel Resistance in Clopidogrel SAPT

Among 145 patients who had recurrent ischemic stroke and were receiving clopidogrel SAPT, 66 (45.5%) showed clopidogrel resistance. Those with clopidogrel resistance had a higher prevalence of hyperlipidemia (*p* = 0.018). The prevalence of smoking was lower in those with than in those without clopidogrel resistance (*p* = 0.022). The multivariate analysis revealed that hyperlipidemia was associated with clopidogrel resistance (odds ratio [OR] = 2.625, 95% CI = 1.187–5.805; *p* = 0.017). Smoking had a protective effect against clopidogrel resistance (OR = 0.426, 95% CI 0.210–0.861, *p* = 0.018, Table 2).

**Table 2:** Factors associated with clopidogrel resistance after clopidogrel SAPT.

	**Clopidogrel Resistance (–) (*n* = 79)**	**Clopidogrel Resistance (+) (*n* = 66)**	***P-value***	OR^*^	**95% CI**	***P-value***
Age, years	70.0 (10.3)	69.3 (11.6)	0.712			
Male	49 (62.0)	38 (57.6)	0.586			
Hypertension	72 (91.1)	59 (89.4)	0.723			
Diabetes mellitus	34 (43.0)	34 (51.5)	0.308			
Hyperlipidemia	50 (63.3)	52 (81.3)	0.018	2.625	1.187–5.805	0.017
Smoking	40 (50.6)	21 (31.8)	0.022	0.426	0.210–0.861	0.018
History of CAD	7 (8.9)	9 (13.6)	0.361			
BMI (Kg/m^2^)	25 (4.0)	25 (3.6)	0.972			
Laboratory findings						
C-reactive protein (mg/L)	0.9 (2.4)	1.3 (2.9)	0.436			
Hemoglobin A1c (%)	6.6 (1.5)	6.5 (1.3)	0.766			

*Results are expressed by number (% column), mean (SD), or median (25–75 percentile range).*

* *Factors entered to model: Dyslipidemia Smoking.*

*CR, clopidogrel resistance; OR, odds ratio; CI, confidential interval; CAD, coronary artery disease; BMI, body mass index; HDL, high-density lipoprotein; LDL, low-density lipoprotein; CRP, C-reactive protein; NIHSS, National Institute of Stroke Scale; mRS, modified Rankin Scale*.

A history of CAD (beta = 0.119; 0.194–0.672; *p* = 0.001) and smoking (beta = −0.315, −0.490–−0.081; *p* = 0.007) was independently associated with a high PRU value.

### Factors Associated With Clopidogrel Resistance in DAPT

Among 131 recurrent ischemic stroke patients receiving DAPT, those with clopidogrel resistance (*n* = 41; 31.5%) were likely to be older (75 ± 8 vs. 69 ± 9 years-old; *p* < 0.001) and female (53.7 vs. 32.6%; *p* = 0.022); they were also likely to smoke less (29.3 vs. 49.4%; *p* = 0.031), low HbA1c (7.1 vs. 6.3%; *p* = 0.001), and have a higher ARU (472 ± 70 vs. 431 ± 74; *p* = 0.004).

From the results of the multivariate analysis, old age (OR = 1.058, 95% CI 1.000–1.118; *p* = 0.048), female sex (OR = 2.465, 95% CI 1.031–5.894; *p* = 0.042), low HbA1c (OR = 0.685, 96% CI 0.489–0.960; *p* = 0.042), and high ARU level (OR = 1.007, 95% CI 1.001–1.013; *p* = 0.018) were independently associated with clopidogrel resistance (Table 3). However, smoking was not significantly associated with clopidogrel resistance in the multivariate analysis.

**Table 3:** Factors associated with clopidogrel resistance after aspirin and clopidogrel treatment.

	**Clopidogrel Resistance (–) (*n* = 89)**	**Clopidogrel Resistance (+) (*n* = 41)**	***p***	OR^*^	**95% CI**	***P-value***
Age, years	68.7 (9.3)	75.0 (8.2)	<0.001	1.058	1.000–1.118	0.048
Male	60 (67.4)	19 (46.3)	0.022	0.406	0.170–0.970	0.042
Hypertension	71 (79.8)	31 (90.2)	0.139			
Diabetes mellitus	45 (50.6)	18 (43.9)	0.480			
Hyperlipidemia	70 (78.7)	33 (80.5)	0.810			
Smoking	44 (49.4)	12 (29.3)	0.031	-	-	*-*
History of CAD	29 (52.7)	11 (45.8)	0.573			
BMI (Kg/m^2^)	24 (3.1)	24 (4.3)	0.432			
Laboratory findings						
C-reactive protein (mg/L)	1.2 (3.1)	2.4 (4.5)	0.121			
Hemoglobin A1c (%)	7.1 (1.7)	6.3 (1.0)	0.001	0.685	0.489–0.960	0.042
Aspirin reaction unit	431 (74)	472 (70)	0.004	1.007	1.001–1.013	0.018

*Results are expressed by number (% column), mean (SD), or median (25–75 percentile range).*

* *Factors entered to model: age, sex, smoking, ARU, and CRP.*

*CR, clopidogrel resistance; OR, odds ratio; CI, confidential interval; CAD, coronary artery disease; BMI, body mass index; NIHSS, National Institute of Stroke Scale; mRS, modified Rankin Scale*.

### Factors Associated With the PRU Level

**Figure 2:**
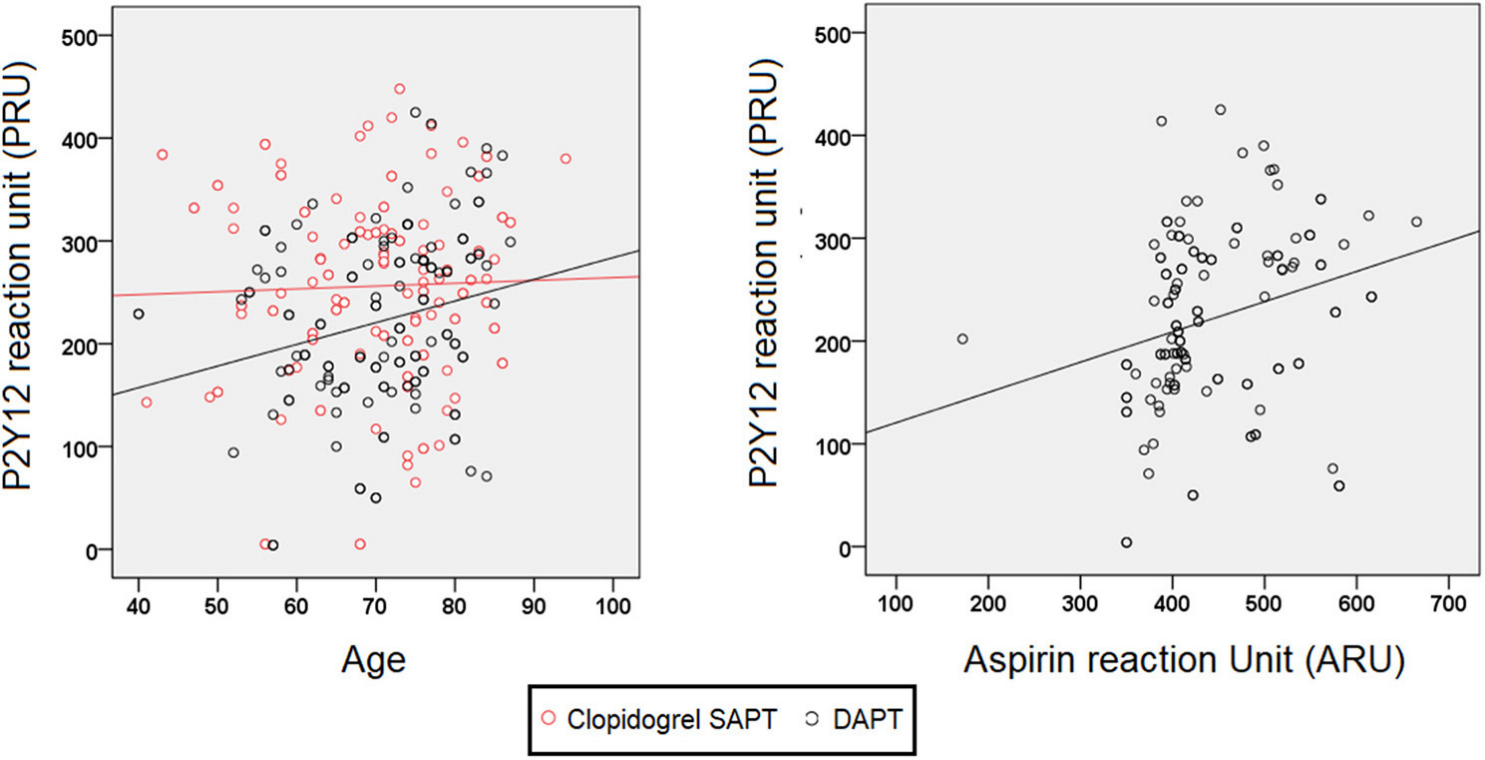
PRUs stratified by age and ARU in recurrent stroke patients receiving DAPT.

The factors associated with the PRU level were age (beta = 0.014, 95% CI 0.004–0.025; *p* = 0.010) and ARU (beta = 0.002, 95% CI 0.001–0.004; *p* = 0.001; Figure 2), but not smoking. PRU increased with age in those receiving DAPT (Pearson *r* = 0.235, *p* = 0.007), but not in those receiving clopidogrel SAPT (Pearson *r* = 0.033, *p* = 0.693; Figure 2). Among those receiving DAPT, the ARU was significantly correlated with PRU (Pearson *r* = 0.261, *p* = 0.003) and percent inhibition (Pearson *r* = −0.292, *p* = 0.001; Figure 2).

## Discussion

In this study, HPR was more frequently observed in recurrent ischemic stroke patients receiving clopidogrel SAPT than in those receiving DAPT. Smoking was independently associated with low PRU and less clopidogrel resistance in recurrent ischemic stroke patients receiving clopidogrel SAPT, but not in those receiving DAPT. Instead, old age, female sex, low HbA1c, and high ARU were independent risk factors for clopidogrel resistance in recurrent ischemic stroke patients receiving DAPT. Age and the ARU level were associated with high PRU level.

Based on our result, HPR and biochemical clopidogrel resistance in smokers may help explain the mechanism underlying the smoker's paradox for clopidogrel. However, the smoker's paradox is still controversial. Smoking decreased short-term and in-hospital mortalities in several studies on CAD (14, 15). However, in long-term studies, a marked increase in long-term mortality negated the positive short-term outcomes (16). The *post-hoc* analysis of the CHANCE trial showed that the smoker's paradox may be observed in ischemic stroke patients receiving short-term DAPT (21 days) during the acute phase (6). However, whether the smoker's paradox will be observed after long-term DAPT in ischemic stroke patients is still unclear. Our results showed that smoking status did not determine HPR or clopidogrel resistance in recurrent ischemic stroke patients after long-term DAPT. Therefore, the smoker's paradox observed in acute ischemic stroke patients receiving short-term DAPT may differ from that observed in those receiving long-term DAPT.

According to treatment guidelines, DAPT is not routinely recommended for the secondary prevention of ischemic stroke (17). Therefore, most patients receive SAPT during the chronic stage of ischemic stroke (18). However, the guidelines for selecting the effective agent for SAPT for long-term secondary stroke prevention are insufficient. Our results showed that HPR was more observed in those with recurrent stroke receiving clopidogrel SAPT than those receiving DAPT. Therefore, when selecting an agent for long-term SAPT after DAPT, considering the factors affecting HPR may be important. In our study, more than 40% of the patients were still smoking at the time of stroke recurrence. In those receiving clopidogrel SAPT, the proportion of smokers was higher in those without than in those with clopidogrel resistance. Smoking was also an independent factor protective against clopidogrel resistance. Therefore, clopidogrel may be considered as a reasonable candidate for long-term secondary stroke prevention in those who fail to quit smoking.

Clopidogrel resistance was less observed in those receiving DAPT than in those receiving clopidogrel SAPT. Clopidogrel resistance may be a more important factor which determines the recurrence of stroke under the use of clopidogrel SAPT then DAPT. In the other hand, mechanisms other than HPR, such as a hemodynamic mechanism may have at least partially influenced the recurrence of stroke under DAPT. Factors associated with HPR also showed some differences between the two groups; hyperlipidemia and smoking status, which are well-known factors associated with HPR under clopidogrel, were associated with clopidogrel resistance in the SAPT group (19), whereas age, female, Hba1c level and ARU was associated with clopidogrel resistance in DAPT group. Age, and high ARU levels are also well-known risk factors of HPR under clopidogrel (20). As the resistance to aspirin was independently associated with clopidogrel resistance, a more common mechanism influencing HPR may have been involved in clopidogrel resistance in recurrent ischemic stroke patients under DAPT.

This study has several limitations. First, this study may have suffered from selection bias as it was retrospective. However, we have tried to minimize this by consecutively including recurrent ischemic stroke patients visiting to each center. For the same reason, we cannot have analyzed genetic testing (such as CYP2C19 loss of function). However, this study analyzed the retrospective data in “actual clinical practice.” Tests related to CYP2C19 LOF alleles are generally not used in clinical practice. Second, it was impossible to analyze the timing of smoking cessation and the exact period of long-term DAPT before the recurrent stroke event due to the retrospective nature of this study. However, we attempted to determine smoking status by comparing past and recent records as soon as possible. Third, the information regarding previous strokes was more often diagnosed at different hospitals where it was first diagnosed. Therefore, accurate information about this was not available. Finally, we could not show the difference between stroke recurrence in smokers and non-smokers receiving long-term clopidogrel treatment. A well-designed study focusing on this may be needed.

We demonstrated that HPR is more frequent in recurrent stroke patients receiving clopidogrel SAPT than in those receiving DAPT. The rates of HPR and clopidogrel resistance were lower in current smokers. The authors believe that smoking is a major risk factor for ischemic stroke, and smoking cessation is necessary (21–24). However, we argue that it may be beneficial to consider the factors affecting HPR, such as smoking status, when selecting the SAPT agent for long-term secondary stroke prevention.

## Data Availability Statement

The original contributions generated for this study are included in the article/supplementary material, further inquiries can be directed to the corresponding author/s.

## Ethics Statement

The studies involving human participants were reviewed and approved by the institutional review board of Jeonbuk National University Hospital (approval number: CUH 2020-01-008). The ethics committee waived the requirement of written informed consent for participation.

## Author Contributions

HK and BK contributed to the design of this study and collected the raw clinical data. HK, SL, SH, D-iC, and BK contributed to the analysis of data, computational studies, and writing of the manuscript. HK contributed to this work as the first author. All authors read and approved the final manuscript.

## Conflict of Interest

The authors declare that the research was conducted in the absence of any commercial or financial relationships that could be construed as a potential conflict of interest.

## Publisher's Note

All claims expressed in this article are solely those of the authors and do not necessarily represent those of their affiliated organizations, or those of the publisher, the editors and the reviewers. Any product that may be evaluated in this article, or claim that may be made by its manufacturer, is not guaranteed or endorsed by the publisher.
